# Bridging the gap between tumor and disease: Innovating cancer and glioma models

**DOI:** 10.1084/jem.20220808

**Published:** 2024-12-03

**Authors:** Stefano M. Cirigliano, Howard A. Fine

**Affiliations:** 1 Department of Neurology, Weill Cornell Medicine, New York, NY, USA; 2 Meyer Cancer Center, Weill Cornell Medicine, New York, NY, USA

## Abstract

Recent advances in cancer biology and therapeutics have underscored the importance of preclinical models in understanding and treating cancer. Nevertheless, current models often fail to capture the complexity and patient-specific nature of human tumors, particularly gliomas. This review examines the strengths and weaknesses of such models, highlighting the need for a new generation of models. Emphasizing the critical role of the tumor microenvironment, tumor, and patient heterogeneity, we propose integrating our advanced understanding of glioma biology with innovative bioengineering and AI technologies to create more clinically relevant, patient-specific models. These innovations are essential for improving therapeutic development and patient outcomes.

## Teaser

Cirigliano and Fine explore the past, present, and future of glioma modeling, identifying limitations in current models and proposing new approaches integrating bioengineering and artificial intelligence. These innovations aim to better capture tumor heterogeneity and human brain microenvironment, enhancing therapeutic relevance and clinical translatability.

## Introduction

In the last decade, there have been unprecedented advances in our understanding and ability to treat cancer. Preclinical models of cancer have played a pivotal role in these advances as a cornerstone tool of cancer research. Nevertheless, despite the explosion of new cancer biology through advances in multiomics technologies and the advent of highly novel therapeutic strategies such as immunotherapy and precision medicine, with some notable exceptions, preclinical cancer modeling has not substantially changed in the last couple of decades (Garraway and Lander, 2013). This raises the question of whether our legacy models are optimally constructed to represent the new biology and to screen for the next generation of innovative therapeutics.

There have been numerous excellent and comprehensive reviews of cancer modeling previously published, and this article will not attempt to review the field as a whole (Ben-David et al., 2019; Akter et al., 2021; Antonica et al., 2022; Tuveson and Clevers, 2019; Ventura and Dow, 2018; Veninga and Voest, 2021; Tuveson and Clevers, 2019; Simian and Bissell, 2017; Smith and Tabar, 2019; Sajjad et al., 2021; Lai et al., 2017; Sampetrean and Saya, 2018; Kersten et al., 2017; Lenting et al., 2017; Ben-David et al., 2019; Morgan et al., 2016; Breitenbach and Hoffmann, 2018; Ogilvie et al., 2017; Lampreht Tratar et al., 2018; Klinghammer et al., 2017; Antonica et al., 2022; Zuckermann et al., 2015; Erices et al., 2023; Mulder et al., 2023; Winkler et al., 2023; Suvà and Tirosh, 2020; Gimple et al., 2022; Ren et al., 2023; Frederico et al., 2023; Hicks et al., 2021a, 2021b; Xu et al., 2021; Da Hora et al., 2019). Instead, we will discuss where the current state of cancer modeling resides within the context of the changing face of cancer research and therapeutic development. We will use glioblastoma (GBM), one of the most therapeutically intractable and lethal human cancers, as a representative case for highlighting the strengths and weaknesses of these models. In doing so, we propose the central hypothesis that current glioma models excel at elucidating specific reductionist views of cancer biology but largely fail in therapeutic screening due to their inability to capture the multifaceted and patient-specific nature of human cancers—for, in fact, cancer is not a cell-autonomous disease. We argue that the next generation of preclinical tumor models will need to be highly contextualized based on the specific tumor type and biological and clinical phenotypes unique to host variables.

## Glioma models: Past and present

The earliest attempts to cultivate human glioma tissue in vitro date back to the early 20th century. A notable effort came in 1924 when Cushing and Bailey successfully maintained small sections of glioma tissue in plasma clots for several days. During this time, they observed cell division and growth. This approach, commonly referred to as the “hanging drop technique,” enabled researchers to conduct short-term studies of viable glioma cells in an external environment (Gómez-Oliva et al., 2021).

In the years that followed, researchers refined these methods, seeking to improve cell survival and study conditions. In 1933, Russell and Bland developed a more advanced system using roller tube cultures, which allowed glioma cells to survive for longer periods (Gómez-Oliva et al., 2021). Then, in 1941, Zimmerman and Arnold further modified the hanging drop method by positioning glioma cells in plasma clots on glass coverslips, which facilitated detailed microscopic analysis of the cells (Ledur et al., 2017). Despite the challenges of these early techniques, particularly the limited ability to maintain long-term cell cultures, they laid the groundwork for even more sophisticated methods for studying glioma cell behavior in the laboratory (Paolillo et al., 2021).

The late 1960s and early 1970s witnessed the development of immortalized glioma cell lines. Notably, the human U87 cell line was established in 1968, providing a readily available, easy-to-use, and continuously replicating model for laboratory studies (Pontén and Macintyre, 1968). Carcinogen-induced rodent tumor cell lines like C6 and 9L were also introduced during this period (Allen et al., 2016; Post and Dawson, 1992). While these cell lines became staples in glioma research due to their practicality and cost-effectiveness, they lacked genomic and transcriptomic similarity to primary human gliomas and did not replicate key clinical features such as central nervous system (CNS)–specific growth and diffuse brain infiltration (Torsvik et al., 2014; Stylli et al., 2015).

As tractable and easy to use are in vitro cell lines, in vivo tumor models have historically represented a level of complexity and clinical relevance far beyond those in the laboratory although that is slowly changing (see below). In general, in vivo models can be divided into syngeneic and xenogeneic models. Besides humans, only dogs develop spontaneous gliomas, and although they bear a very close genetic, pathologic, and biological resemblance to human gliomas, canine GBMs are more rare than human gliomas, limiting their experimental use (Hicks et al., 2021b). Carcinogen-induced gliomas have been experimentally produced in a number of different animal specials ranging from drosophila to mice and even Rhesus monkeys (radiation-induced), but have limitations as models given their unpredictable tumor penetrance, timing, and lack of genomic similarity to human gliomas (Sampson et al., 1997; Oh et al., 2014; Wouters et al., 2020; Lonser et al., 2002).

The advent of the genetic and molecular biology age in the 1980s ushered in a dramatic advance in human tumor modeling with the introduction of genetically engineered mouse models (GEMMs) of human cancer. These models allowed for the manipulation of specific oncogenic-associated genes to facilitate the identification of genomic drivers and signaling pathways involved in gliomagenesis. These models, however, were not without limitations for they were costly, exhibited long latencies, variable tumor penetrance, and generally lacked the genomic complexity of human gliomas, making them less suitable for high-throughput drug screening (Kersten et al., 2017; Figg et al., 2024; Hicks et al., 2021b; Stylli et al., 2015).

Prior to GEMMs, xenograft models using legacy glioma cell lines, though cost-effective, lacked molecular and genetic similarity to human GBM (Richmond and Su, 2008; Finkelstein et al., 1994). A major advance came with orthotopic patient-derived xenografts (PDXs), which better replicate the genomics of the original human tumor. PDXs can be derived either from tumor fragments or glioma stem cells (GSCs). Transplanting human GBM tissue directly into immunodeficient animal brains preserves genomic complexity and heterogeneity by minimizing in vitro clonal selection (Kerstetter-Fogle et al., 2020; Vaubel et al., 2020; Lai et al., 2017). Nevertheless, challenges remain, including clonal selection in vivo and difficulty retaining tumor characteristics in the mouse brain (Pine et al., 2020). Moreover, PDX tumors often grow as dense, relatively isolated masses, unlike infiltrative human gliomas, limiting drug penetration studies and excluding meaningful analysis of immune interactions due to the immunodeficient host environment.

The next advancement in creating more clinically relevant GBM models occurred in the early 2000s with the emergence of tumor/GSCs, which offered a model that better replicated primary tumor properties, including in vivo brain invasion (Lee et al., 2006; Suvà and Tirosh, 2020; Venere et al., 2011). GSCs have been shown to better resemble the genomic landscape of the parental tumor and display greater genomic stability on serial passage. Importantly, and in contrast to most legacy glioma cell lines and many tumor chunk-derived PDXs, GSC form highly infiltrative tumors in orthotopic PDXs in a manner highly similar to that seen in the brains of patients with GBMs (Lee et al., 2006). Nevertheless, despite their superiority in mimicking tumor behavior, GSCs are difficult to work with due to variable growth rates and penetrance and are relatively resource-intensive leading many researchers (and pharmaceutical/biotechnology companies) to continue using legacy cell lines for convenience and cost.

In the past decade, significant progress has been made in developing more tractable in vitro and ex vivo glioma models that incorporate aspects of in vivo modeling and the tumor microenvironment, such as novel organotypic models. A key limitation of in vitro models is their inability to capture the complex tumor–host interactions, while in vivo models, though more physiologically relevant, face challenges in non-human contexts, cost, and limited experimental flexibility. Tumor/glioma organoids—3D growths of primary glioma cell lines or GSCs—provide a closer approximation to human gliomas but lack interactions with normal brain cells (Hubert et al., 2016). Organotypic glioma models, including brain slice cultures, enable glioma cells to interact with normal brain cells and extracellular matrix. Patient-derived glioma slices offer an improved tumor microenvironment model, though they are limited by stress and the eventual death of normal brain constituents in vitro (LeBlanc et al., 2022; Eisemann et al., 2018; Marques-Torrejon et al., 2018).

New and promising patient-derived human brain-glioma models, based on advanced 3D tissue and organoid engineering technologies, are being developed and have already demonstrated significant potential in replicating patient tumors with unprecedented accuracy. Notably, researchers have created GBM organoids (GBOs) from 1-mm tumor chunk explants (Jacob et al., 2020). These GBOs are cultured under serum-free conditions, without added growth factors or extracellular matrix components, allowing them to maintain the native cytoarchitecture and cell interactions found in the original tumors. Single-cell transcriptomics has confirmed the preservation of both tumor and non-tumor cell populations over 2 wk of culture, underscoring the model’s robustness in reflecting the diverse cellular composition of GBMs (LeBlanc et al., 2022). Recent spatial transcriptomics studies have further underscored the complexity of GBM tissue architecture, revealing a layered cellular organization influenced by hypoxia. To better emulate these spatial gradients, microfluidic systems like the “GBM-on-a-chip” are particularly promising. This model incorporates brain-like vascular growth, immune cells, and tumor cells within a multiregion setup, utilizing an oxygen gradient and an hyaluronic acid (HA)–rich Matrigel ECM to replicate the complexity of the tumor microenvironment (Cui et al., 2020).

To better recapitulate the human brain microenvironment within a sustainably viable context, human embryonic or induced pluripotent stem cell–generated cerebral organoids have been genetically manipulated to develop spontaneous oncogenic properties or co-cultured with GSCs. This latter approach, known as glioma cerebral organoid (“GLICO”), allows for the diffuse infiltration of glioma cells into a human brain–like microenvironment, as occurs clinically with high-grade gliomas (da Silva et al., 2018; Linkous et al., 2019). These models can closely recapitulate the genomic heterogeneity and epigenomic-mediated transcriptomic cellular states of a given patient’s primary tumor and enable high-throughput patient-specific drug screening (Pine et al., 2020, 2023). Such models, however, still face limitations in their inability to replicate immune–tumor cell interactions and vascular dynamics, although early attempts to reconstitute an in vitro immune niche in these organoid models appear promising (Polak et al., 2024).

Currently, efforts are focused on developing models that more accurately recapitulate the human brain microenvironment in a sustainable context, incorporating elements such as an intact immune system and vascular components, as will be discussed in detail in the following sections. Reflecting this shift, the Food and Drug Administration has recently moved away from requiring animal testing before human drug clinical trials (Adashi et al., 2023). This change aligns with advancements in alternative preclinical models, including organoids, organ-on-a-chip systems, and other in vitro methods, which are increasingly recognized as more human-relevant and provide predictive data for human responses without relying solely on animal models. Table 1 provides an overview of the different types of glioma models, while Table 2 lists the majority of published models to date and their basic characteristics.

**Table 1.** Overview of current pre-clinical models to study gliomas

Model	Method	Name/aliases	Origin	Characteristics	Growth condition/maintenance	Main feature	Limitations	Citations
In vitro	Immortalized adherent cell lines	Established/traditional/classic cancer cell lines	Enzymatic disaggregation of isolated tumor explants into individual cells with the capacity to proliferate indefinitely in the presence of fetal bovine serum and defined nutrients. Cancer cell lines constitute one of the most common cancer models due to their simplicity, inexpensiveness, and high proliferation rates.	High proliferation rates, harbor genotypes somewhat similar to primary tumor (e.g., TP53, PTEN).	Media with serum fetal bovine	Accessibility, reproducibility	Genomically unstable, deficient in many/most properties of human gliomas (e.g., invasiveness in vivo).	Richter et al. (2021)
	Glioma cancer stem-like cells	GSCs, TICs, CSCs	Freshly resected and dissociated GBM tissue.	Self-renewal capacity, genetically stable upon multiple passages, more closely resembles primary tumors, retains patient’s tumor subtype.	Neurobasal media with B27, EGF, and FGF	Phenocopy patient’s transcriptomic subtype	Cycling cells overrepresented, more difficult and expensive to grow than legacy glioma lines.	Lee et al. (2006)
	3D tumor spheroids/organoids	Cancer tumor organoids, CSC organoids	Primary-derived GSCs, explants from xenograft models, genetically engineered glioma cells, or direct patient samples.	Self-organization, differentiation, increased cell diversity, phenotypically diverse populations.	Scaffold ECM like Matrigel or hanging drop, ultra-low round-bottom plates	Hypoxic gradients	Does not recapitulate the microenvironment of the brain or glioma-host cell, glioma brain ECM interactions.	Hubert et al. (2016)
Ex vivo	Co-cultures of hESCs-derived mini-brains and GBM stem cells	GLICOs	hPSCs-derived cerebral organoids co-cultured with GSCs.	Mimic various aspects of the human microenvironment, preserve native cytoarchitecture and cell interactions, intratumor heterogeneity.	Cerebral organoid media	Human tumor–host interactions	Lacks spatial patterns of tumor heterogeneity and is devoid of vascular or immunologic components.	Linkous et al. (2019)
	Tumor explants	PDOs, GBM organoids	1-mm tumor chunk explants cultured in serum-free conditions and without added growth factors or extracellular matrix.	Amenable for high-throughput screening, preserve some tumor microenvironment (TME).	Growth factors-free chemically defined medium	Maintain original patient’s TME	Inter-patient variable efficiency, tissue starts dying almost immediately so system under stress, thus best for short-term assays.	Jacob et al. (2020)
	Microfabrication techniques	GBM-on-a-chip	Multi-region layout for brain vascular growth, immune cell seeding, and tumor growth, as well as a central area for cell culture medium infusion.	Allow an oxygen gradient to simulate the role of hypoxia in GBM tissue organization. Further enhancement of its physiological relevance.	Brain-mimicking, HA-rich Matrigel ECM	Spatial heterogeneity	Artificial and relatively simplistic constitution of TME, low-throughput scale.	Cui et al. (2020)
In vivo	Syngeneic: Spontaneously and carcinogenic	Spontaneous tumor, carcinogen-induced model	Tumor cells implanted into immunocompetent mice of the same genetic background, through (1) the use of carcinogens to induce tumorigenesis (either in vitro or in vivo) or (2) by leveraging spontaneous tumor formation in dogs.	Immunogenic, suitable for immunotherapy studies.	Serial transplantation or passaging in vitro; naturally occurring in the case of dogs	Immunogenecity	In the case of dogs, rare and difficult to obtain. Spontaneous tumors of non-human origin are not generally reflective of the GBM genome.	Oh et al. (2014)
	Syngeneic: GEMM	GEMM	By inducing tumor formation through targeted modifications to the mouse genome. Combination of at least two or more genetic events, such as alteration in PTEN, NF1, TP53, KRAS, EGFR, and PDGF β on immune-competent mice.	Useful model to study gliomagenesis.	Serial transplantation or passaging in vitro	Recapitulates tumor initiation	Mouse tumor origin, gene-driven tumorigenesis with limited heterogeneity, mouse brain microenvironment.	Richmond and Su (2008)
	Xenografts: Orthotopic	PDX	Hetero-transplantation of human cancer cells into immune-deficient mouse strains such as nude mice, non-obese diabetic mice, severe combined immunodeficient mice.	Traditional cell lines typically form well-demarcated, solid-like tumors. In contrast, GSCs and PDX more closely mimic the infiltrative growth patterns seen in human gliomas.	Patient-derived, GSCs cells injected into the brain parenchyma	Gold standard for tumorigenicity test	Mouse TME, lack of immune cells.	Sarkaria et al. (2006)
	Xenografts: Heterotopic	PDX	By implanting glioma cells subcutaneously. Engraftment success rates can be notably enhanced.	Quick growth, easily scalable.	Legacy glioma cell lines (not GSCs) injected subcutaneously	Better engraftment	Non-brain TME, true GSCs do not grow well in a microenvironment other than the CNS.	Akter et al. (2021)
	Zebrafish xenograft models	Zebrafish PDX	Zebrafish embryos.	Transparent nature in early developmental stages, genetic and anatomical similarities with humans.	Maintained in standard zebrafish facility conditions	Rapid development, cost-effective, high-throughput drug screening	Temperature regulation, differences in pharmacokinetics and pharmacodynamics. Do not recapitulate GBM biology, genomics, or human TME.	Alberti et al. (2024)

TIC: tumor-initiating cells, CSC: cancer stem cells, hESC: human embryonic stem cell, shPSC: human pluripotent stem cells, EGF: epidermal growth factor, FGF: fibroblast growth factor, MGMT: O(6)-methylguanine-DNA methyltransferase, TMZ: temozolomide.

**Table 2.** Comprehensive table with examples of most the published pre-clinical glioma models and their specifics over the last 30+ years

Model	Origin	Name	Characteristics	Citations
Established cell lines	Human GBM	U87	High proliferation, non-diffuse, infiltrative patterns, widely used, well-characterized	Clark et al. (2010), Allen et al. (2016)
Established cell line	Human GBM	U251	High proliferation, necrotic regions, high Ki-67 positivity, maintains some tumor cell infiltrative patterns	Torsvik et al. (2014), Li et al. (2017)
Established cell line	Human GBM	T98G	High ACTA2 expression, motility, minimally tumorigenic in mice	Kiseleva et al. (2016), Rubenstein et al. (1999)
Established cell line	Human GBM	A172	High proliferation	Fenstermaker et al. (1998), Kiseleva et al. (2016)
Established cell line	Human GBM	LN229	High proliferation, useful for studying MGMT methylation and drug response	Beckner et al. (2005), Demircan et al. (2021)
Established cell line	Human GBM	SF8628	H3.3K27M mutation, used in pediatric glioma studies	Damanskienė et al. (2022), Olow et al. (2016)
Established cell line	Human GBM	U373	High proliferation, widely used, well-characterized	Dranoff et al. (1985), Takiguchi et al. (1985)
Established cell line	Human GBM	SF9402	H3.3 wild type, used in combination therapy studies	Hashizume et al. (2014), Wang et al. (2021)
Established cell line	Human GBM	SF7761	H3.3K27M mutation, used in pediatric glioma studies	Hashizume et al. (2012), Abe et al. (2020)
Established cell line	Human GBM	GBM12	High proliferation, used in xenograft studies	Sarkaria et al. (2006), Paraskevakou et al. (2007)
Established cell line	Human oligodendroglioma	Hs683	High proliferation, useful for studying TMZ response	Tasiou et al. (2001), Konduri et al. (2001)
Established cell line	Mouse GBM	GL261	High MHC I, MHC II expression, RAS and p53 mutations. Widely used in immunotherapy studies	Wu et al. (2008), Rappa et al. (2008)
Established cell line	Rat gliosarcoma	9L	High proliferation, aggressive tumor growth in vivo	Ghods et al. (2007), Kruse et al. (1994)
Established cell line	Rat GBM	C6	High proliferation, aggressive tumor growth in vivo	Giakoumettis et al. (2018), Kondo et al. (2004)
3D models	Human GBM	GSCs	Retains patient’s molecular subtypes	Singh et al. (2004), Lee et al. (2006)
3D models	Human GBM	PDOs	Recapitulates inter- and intra-tumoral heterogeneity. Requires fresh tumor samples	Chen et al. (2022), Jacob et al. (2020)
3D models	Human GBM	GLICOs	Allows for diffuse infiltration of glioma cells, mimicking human brain integration. Represents epigenomic-mediated transcriptomic states	da Silva et al. (2018), Linkous et al. (2019)
3D models	Human GBM	Cerebral organoids with CRISPR-Cas9 (GBM organoids)	Genetically manipulated to develop oncogenic properties. Limited tumor heterogeneity	Ogawa et al. (2018), Bian et al. (2018)
3D models	Human GBM	Tumor organoids	Represents hypoxic niches and oxygen gradients. Lack interactions with normal host brain cells	Hubert et al. (2016), Heaster et al. (2019)
3D models	Human and rodent brain tissue	Brain slice cultures (explants)	Allows interaction with normal brain cells and ECM. Limited by the stress and eventual death of normal brain constituents	Ohnishi et al. (1998), Eisemann et al. (2018)
Oncogene induced syngeneic mouse models	C57BL/6 mouse	SB28	Poor immunogenic glioma model, commonly used in PD-L immunotherapy studies	Letchuman et al. (2022), Murty et al. (2020)
Chemically induced syngeneic mouse models	C57BL/6 mouse	GL261	High proliferation, aggressive tumor growth, mutation in KRAS. Genetic drift over time, limited representation of human gliomas, immunogenic.	Szatmári et al. (2006), Daviaud et al. (2024)
Chemically induced	C57BL/6 mouse	CT-2A	Deficient in PTEN, necrotic, chemoresistant, undergoes unregulated angiogenesis. Used in immunotherapy studies limited genetic heterogeneity	Riva et al. (2019), Casanova-Carvajal et al. (2019)
Chemically induced	C57BL/6 mouse	GL26	High proliferation, mutation in KRAS, aggressive tumor growth, widely used	Wouters et al. (2020), Kim et al. (2010)
Spontaneous syngeneic models	C3H mouse	P560	High proliferation, aggressive tumor growth	Kim et al. (2010), Bradford et al. (1986)
GEMMs	Neural progenitors or astrocytes	Ink4a-Arf/Kras/Akt	Model for studying cooperation between KRas activation and Ink4a-Arf loss in gliomagenesis	Uhrbom et al. (2002), Figg et al. (2024)
GEMMs	Various glioma-initiating cells	PTEN/p53/CDKN2/RB knockout	Models high-grade astrocytomas with multiple genetic alterations	Holland (2001), Alcantara Llaguno et al. (2019)
GEMMs	Neural stem/progenitor cells	NF1/p53	Useful for studying NF1 and p53 interactions in gliomagenesis. Induces tumors that appear similar to astrocytomas	Zhu et al. (2005), Liu et al. (2011)
GEMMs	Various glioma-initiating cells	Idh1R132H	Investigates metabolic vulnerabilities of IDH1 mutant gliomas. Demonstrates extreme vulnerability to NAD+ depletion	Bardella et al. (2016), Zhang et al. (2019)
GEMMs	Various glioma-initiating cells	CDKN2 Knockout/EGFR/PDGFR	Studies cooperation between CDKN2 loss and growth factor signaling in gliomagenesis	Zhu et al. (2009), Yeo et al. (2021)
Xenograft models	Human GBM	U87MG xenograft	High proliferation, circumscribed tumors. Widely used, good reproducibility. Lacks infiltrative pattern seen in human gliomas	Mathieu et al. (2008), Donoghue et al. (2011)
Cell line xenograft	Human GBM	U251 xenograft	High proliferation, necrotic regions, well-characterized, widely used, limited heterogeneity, circumscribed tumors	Williams et al. (1998), Kijima et al. (2014)
Cell line xenograft	Human GBM	A172 xenograft	High proliferation, used in various glioma studies, limited heterogeneity	Finkelstein et al. (1994), Zhang et al. (2008)
LN229 xenograft	Human GBM	LN229 xenograft	High proliferation, MGMT methylation	Hlavaty et al. (2011), Nagai et al. (2023)
SF8628 xenograft	Human GBM	SF8628 xenograft	H3.3K27M mutation, used in pediatric glioma studies	Olow et al. (2016), Da-Veiga et al. (2023)
IDH1 mutant xenograft	Human GBM	IDH1mut xenograft	Retains IDH1 mutation, mimics genetic and phenotypic features of primary tumors. Challenging to maintain	Luchman et al. (2012), Rudà et al. (2024)
EGFRvIII xenograft	Human GBM	GLI36-EGFRvIII xenograft	Overexpression of EGFRvIII, aggressive tumor growth	Herrmann et al. (2016), Saydam et al. (2005)
Xenograft	Human GBM	GSC xenograft	CD133^+^ cells, tumor-initiating capability. Preserves tumor heterogeneity. Long latency periods	Lee et al. (2006), Tanaka et al. (2019)
Xenograft	Human GBM	PDX	Retains some genetic and histological features of the primary tumor. Preserves tumor heterogeneity. Requires fresh tumor samples with variable success rates	Kerstetter-Fogle et al. (2020), Vaubel et al. (2020)

## Modeling cancer’s biological complexity

**Figure 1.**
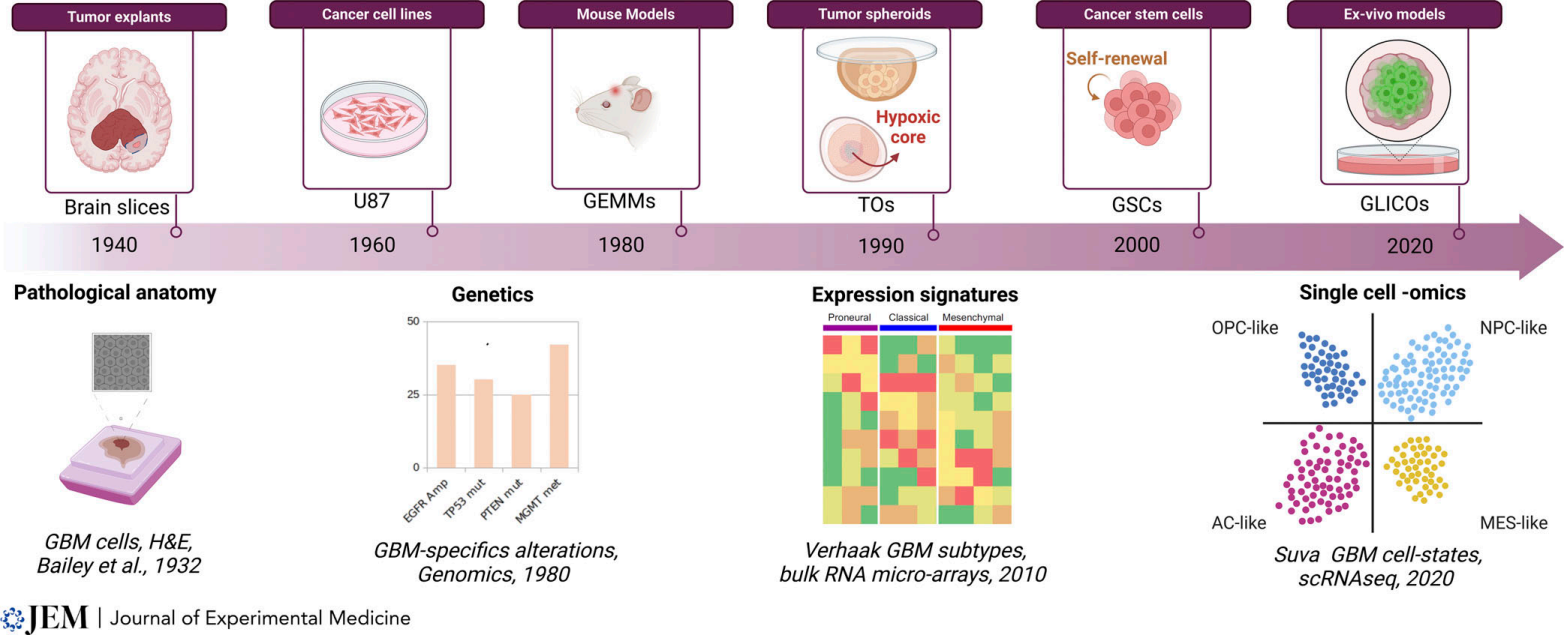
**Timeline of GBM preclinical modeling.** The timeline traces key developments in GBM modeling, starting with tumor explants in the 1940s, cancer cell lines in the 1960s (e.g., U87), GEMMs in the 1980s, tumor organoids/spheroids (TOs) in the 1990s, cancer stem cells (GSCs) in the 2000s, and ex vivo models like GLICOs in the 2020s. Examples of notable landmarks driving these models include the identification of pathological anatomy in the 1930s (Bailey and Eisenhardt, 1932), genetic alterations in the 1980s (McDonald and Dohrmann, 1988), gene expression signatures in 2010 (Verhaak et al., 2010), and single-cell transcriptomics in the 2020s (Neftel et al., 2019). scRNAseq: single-cell RNA sequencing, OPC: oligodendrocyte progenitor -like cell, NPC: neural progenitor -like cell, AC: astrocyte -like cell, MES: mesenchymal-like cell. Created in BioRender.

Over the past three decades, our understanding of cancer pathogenesis has expanded significantly, with models playing a crucial role (Fig. 1). This success largely reflects a reductionist approach, focusing on discrete mechanistic processes such as tumor initiation, cell survival, and cell proliferation. These surrogate endpoints of cancer have been instrumental in elucidating genetic and molecular mechanisms of tumor initiation and propagation, as well as in screening traditional cytotoxic and antiproliferative chemotherapeutic agents.

Cancer, however, is not a cell-autonomous disease, but rather one involving complex interactions within its microenvironment and the broader organism, which are not adequately captured by reductionist models. Such models often fail to replicate the intricate interactions and regulatory mechanisms present in native tumor environments, limiting their ability to study complex pathophysiological processes. These limitations hinder our ability to study these complex dynamics operative in cancer patients, can obscure our ability to identify tractable clinically effective novel therapeutic targets clearly, and may impede the development of preclinical screens that more efficiently predict clinically active agents. To address these challenges, it is essential to consider at least three levels of complexity in cancer modeling:

### Level 1: Factors independent of tumor type

Hanahan and Weinberg’s “Hallmarks of Cancer” represent the inherent complex processes found in all cancers (Hanahan, 2022). While individual hallmarks—often those considered cell-autonomous—are fairly well-represented in our current models, the true value of the hallmarks for describing human cancer lies in the multiplicity of hallmark interactions, including both seemingly cell-autonomous as well as non-cell-autonomous processes such as immune evasion, metastasis, invasion, and the microbiome. For instance, while oncogenes cause dysregulation of cellular metabolism, cancer induces profound metabolic changes throughout the entire patient, including a shift toward a systemic catabolic state that results in physiological conditions such as cachexia and immune dysfunction (Fearon et al., 2012; Critchley-Thorne et al., 2009).

Similarly, invasion and metastasis are not purely cancer cell-autonomous functions (Bischof and Irminger-Finger, 2005; Ferretti et al., 2007). The local tissue environment, including factors such as the extracellular matrix and local immune landscape of specific tissues and organs, play crucial roles in a tumor cell’s ability to breach physiological barriers like basement membranes, vasculature, and lymphatics (Iadecola, 2004; Abbott et al., 2006; Liebner et al., 2011). Techniques such as injecting xenografts into normal tissue or the bloodstream artificially disrupt these barriers, failing to model the natural process of clonal selection and tissue-specific interactions like metastasis (Lee et al., 2011). Additionally, the mechanisms used for invasion may differ across species, affecting the relevance of these models to human cancer (Jain et al., 2007).

Even cell-autonomous hallmarks, such as the ability to sustain proliferative signals, are influenced by local tissue factors and systemic host processes. For instance, local angiogenic factors within the ECM, which promote tumor vascularization and alleviate tumor hypoxia, as well as endocrine factors like insulin/insulin-like growth factor and sex hormones (e.g., androgens, estrogens, progesterones), can significantly impact proliferative signaling (Chuffa et al., 2017; LeRoith and Roberts, 2003). Moreover, replicative immortality, though often considered cell-autonomous, can be influenced by host factors, as demonstrated by classic experiments showing malignant melanoma cells behaving normally when injected into the normal tissue of a developing mouse embryo (Mirea et al., 2020). Few cancer cell lines and PDX models adequately capture these organism-level properties of cancer. Even GEMMs often do not recapitulate the spontaneous invasion, metastasis, genomic complexity, and protumorigenic inflammation seen in human cancers (Biegon et al., 2022; Dohi et al., 2008).

### Level 2: Tumor-specific factors—GBM as an example

In addition to general factors intrinsic to most human cancers, the second level of cancer complexity not adequately represented in our models are tumor-specific factors. Using gliomas as a case in point, it is evident that the underpinnings of gliomagenesis adhere to the Hallmarks of Cancer, however, their realization is unique to glioma biology and the human clinical disease of glioma. One of us has recently discussed the uniqueness of GBM compared to other cancers, and although we will not reiterate that discussion in detail here, there are several traits particularly pertinent to the meaningful modeling of malignant gliomas worth mentioning (Fine, 2024).

#### Blood-brain barrier (BBB)

First and foremost is the BBB, a microarchitectural complex of specialized endothelial cells, perivascular stromal support cells, and astrocytes that maintain a selective barrier to the entry of many molecules, pathogens, and cells into the CNS (Liebner et al., 2011). Although there have been attempts to model the BBB in vitro, none have fully succeeded, especially within the context of a glioma-infiltrated brain (Hajal et al., 2022; Williams-Medina et al., 2021). Advocates of in vivo PDXs argue that such models include the BBB; however, the mere act of injecting tumor cells into the brain disrupts, at least transiently, the BBB, initially violating the barrier function and then inducing a long-term traumatic response (e.g., glial scar). Spontaneously forming glioma GEMMs circumvent the BBB and brain trauma issues but involve non-human gliomas, within a non-human brain microenvironment and immune system, making their biological, immunological, and clinical relevance to human gliomas questionable (Yang et al., 2010; Oh et al., 2014).

#### Neural–tumor interactions

Gliomas’ exclusive occurrence in the brain is crucial for clinically relevant glioma modeling for two reasons: first, because these tumors are intertwined with highly sensitive and vital normal brain tissue, and, second, because recent studies have shown that glioma cells are anatomically and functionally connected to other normal brain cells (Winkler et al., 2023; Salvalaggio et al., 2024). These factors significantly influence our understanding of glioma biology and challenge our ability to identify effective treatments. For instance, studies have shown that neurotransmitter-mediated electrical signaling through α-amino-3-hydroxy-5-methyl-4-isoxazolepropionic acid–like synaptic connections between neurons and glioma cells significantly enhances glioma cell survival and proliferation (Krishna et al., 2023; Meyer et al., 2024). A model that does not phenocopy this process misses a significant aspect of human glioma biology. Although human PDXs can form malignant synaptic connections with mouse brain cells, and certain GEMMs make tumor–brain connections, it is well established that synaptic transmission, calcium transients, and neural network connectivity within human brains differ significantly from those seen in mice (Xu et al., 2022; Bakken et al., 2021). Thus, unless human–human glioma–host brain interactions are modeled, we risk missing clinically relevant aspects of glioma biology.

#### Treatment limitations due to brain integration

The interconnected glioma–neuronal network highlights why the effectiveness of surgical and cytotoxic (radiation) therapy for malignant gliomas is constrained by the risk of damaging normal brain tissue, which can result in permanent neurological harm (Turner et al., 2009; Franzini et al., 2019). Unlike other cancers, such as those in the breast or prostate, where tissue destruction may cause cosmetic or functional issues, collateral damage to normal brain tissue during glioma treatment can severely and permanently impair the patient’s quality of life and functionality (Chieffo et al., 2023). Therefore, accurately modeling these interactions in a clinically relevant manner is crucial not only for assessing the potential anti-glioma effects of novel therapies but also for evaluating their potential neurotoxicity.

#### Invasive nature without metastasis

Another defining feature of gliomas is their non-metastatic but highly invasive nature. By the time of diagnosis, almost all high-grade gliomas have diffusely infiltrated and functionally integrated into the surrounding normal brain, making surgical cure impossible (Paolillo et al., 2018). This necessitates the inclusion of large areas of otherwise normal brain intermixed with tumor cells in high-dose radiation fields, which adds potential significant short- and long-term neurological morbidity to treatment (Kagan et al., 1976). Few PDX models truly replicate the degree of glioma invasion seen in patients, thereby minimizing their ability to capture the full extent of the tumoral–host cell functional neural network integration discussed above. In contrast, human cerebral organoid/glioma models replicate the invasive nature and functional integration of GSCs, although they are deficient in key clinical aspects of the invasive process, such as the tendency to spread along myelin-coated white matter tracts and perivascular spaces (Ishihara et al., 2009; Giese et al., 2003).

#### Genomic and epigenomic heterogeneity

Another critically important human glioma characteristic often inadequately modeled is their extensive intratumoral and extratumoral genomic and epigenomic heterogeneity, represented by transcriptomic cellular states (Kinker et al., 2020; Schiffman et al., 2022, *Preprint*). This cellular/clonal heterogeneity allows invading glioma cells to traverse and proliferate in diverse microenvironmental landscapes within the brain and may be instrumental in their intrinsic resistance to therapeutic stress (Nicholson and Fine, 2021). PDX models often fail to mimic this heterogeneity because the foreign microenvironment of a mouse brain may select clones not predominant in the parental human tumor. Evidence for the importance of the human brain microenvironment is demonstrated by the fact that the diversity of GBM isocitrate dehydrogenase–wild type cellular states is better represented in human GLICO-like models than in standard murine vitro or PDX models (Pine et al., 2020, 2023; Wang et al., 2024).

Thus, we believe that models that fail to incorporate most, if not all, of these GBM-unique properties will fall short of representing the full extent of the “disease of GBM” and, although they may still be useful for modeling specific components of GBM biology, will likely be limited in their clinical translatability such as in drug screens for effective therapeutics.

### Level 3: Patient-specific factors

Finally, the third, and least well-modeled level of cancer complexity in our experimental systems are clinical/patient-specific factors. Our current models rarely, if ever, display the diversity of host factors found in patients, such as age, performance status, nutritional status, genomic background, comorbidities, medication, toxin exposure, or prior cancer therapy, all of which profoundly affect cancer induction, maintenance, progression, and response to therapy. This variability in host factors is a major reason for the vast differences in the clinical natural history and therapeutic responses of individual cancer patients (e.g., no anticancer drug has a 100% clinical response rate in any tumor type, and most far less). Indeed, clinical factors such as a patient’s age and performance status have as great an effect on treatment response and overall survival in GBM as many biological variables or treatments (Lawrence et al., 2012; Griazov et al., 2022). No current model systems even attempt to recapitulate this complex, poorly understood, but critically important multifactorial aspect of human disease.

**Figure 2.**
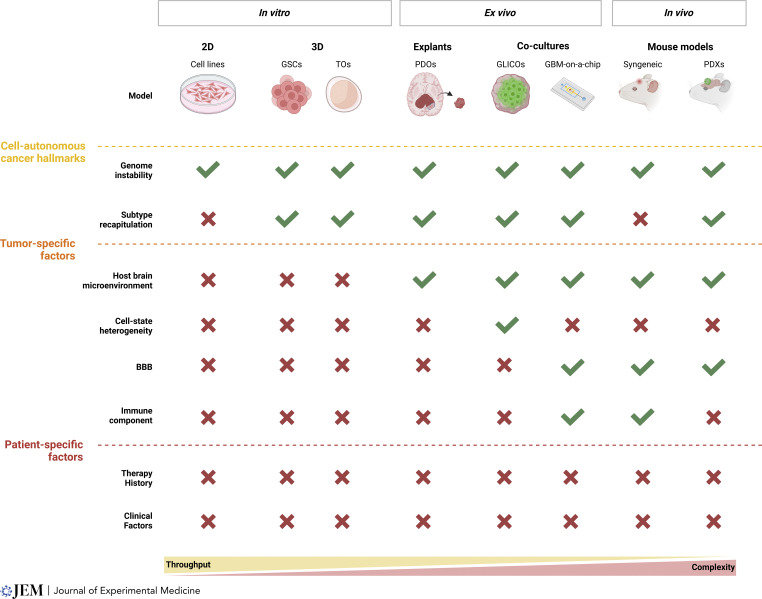
**GBM model throughput, complexity, and cancer hallmarks.** The figure illustrates the incorporation of clinically relevant hallmarks of gliomas and the trade-offs between throughput and complexity across various GBM models: in vitro (2D cell lines, 3D GSCs, TOs), ex vivo (tumor explants, co-cultures like GLICOs and GBM-on-a-chip), and in vivo (syngeneic and PDX mouse models). These models range from cell lines that capture cell-autonomous cancer hallmarks to PDXs and GLICOs that incorporate tumor-specific factors like the BBB and immune components. The most complex future models should account for patient-specific factors, including clinical and treatment history. Created in BioRender.

A detailed discussion of how different models do and do not capture either general cancer or glioma-specific characteristics is beyond the scope of this article; however, Fig. 2 offers an oversimplified but useful framework for understanding which tumor and human cancer characteristics are generally most and least well-represented by each major category of tumor models, fully appreciating the individual exceptions and nuances within each grouping.

In summary, while reductionist approaches and models have advanced our understanding of specific oncogenic mechanisms, they generally fall short of capturing the multifaceted nature of cancer. To truly model human cancer and develop truly predictive preclinical screens, future models will need to integrate the complexity of the tumor microenvironment, patient-specific factors, and systemic host interactions.

## Future modeling

It is useful to think about modeling going forward within the context of how we can best use our current models, what types of improved models can be envisioned over the next several years, and a speculative perspective relative to what such models might look like in the future.

Despite the deficiencies in current glioma models, a number of them are useful within the context of specific and limited questions. For instance, a GEMM created to induce a glioma-like tumor with a constitutively active PIK3CA mutant could be used to evaluate whether a new PI3K inhibitor can interfere with downstream signaling of the pathway and cross an intact and/or partially disrupted (mouse) BBB. One could then use a human glioma PDX harboring such a mutation—within the context of a true human GBM genomic landscape—to ask whether such a molecule similarly interferes with the pathway activation. Through these models, one could assess whether the new drug can cross the BBB and inhibit the pathway within the context of a murine and human glial neuroepithelial tumor. If the answer is no, then it would be fair to conclude that the drug is not likely worthy of further clinical development for gliomas. We would argue, however, that a positive result from these initial experiments should not necessarily be viewed with confidence in the assay’s ability to predict clinical success. Thus, we believe that these models are more useful for rejecting drugs for further development than they are as predictive or even suggestive of clinical success.

Thus, as discussed, our current legacy models are best used for asking contextual and specific biological questions and eliminating potential clinical candidates but are of relatively limited use for distinguishing between the most promising of several potential candidates. Although it is reasonable to hypothesize that congruent results across multiple divergent model platforms (e.g., PDXs, GEMMs, organoids) would increase the probability of selecting a drug that will be successful in the clinic, that assumption has yet to be validated. Going forward, efforts should be made to do just that.

Over the next several years, we believe there are three priorities for new glioma models: first, to develop models that accurately reflect the patient-specific complex genomic, epigenomic, and transcriptomic heterogeneity at the single-cell level found in human gliomas; second, to reproduce the profound intratumoral clonal genomic and epigenomic heterogeneity observed within and between different patients’ gliomas; and third, to incorporate the critically important aspects of the human brain microenvironment, including all of its cellular and noncellular components, into these models (Fig. 2). Failure to achieve these objectives will ultimately result in models lacking clinical relevance, just like our current legacy models. Nevertheless, as briefly discussed, a series of promising new patient-derived human brain-glioma models, based on novel patient-derived 3D tissue and organoid engineering technology, are being developed and have already shown great promise in recapitulating patients’ tumors in ways not previously possible (Cui et al., 2020; Jacob et al., 2020; Pine et al., 2020).

We believe that the next generation of drug screening assays will, at a minimum, incorporate these critical tumor–host interactions while maintaining the simplicity necessary for a tractable and scalable screen. To that end, we acknowledge there is a fundamental tension between developing tumor models that fully capture the genomic, epigenomic, and microenvironmental heterogeneity of GBMs and the pragmatic need for reproducible, stable models, particularly for early-stage proof-of-concept studies and initial drug screening. To reconcile this, we proposed a two-tiered modeling framework. The first tier would consist of biologically validated, genomically stable, and well-characterized models—such as PDXs—for studying tumor vascularization/angiogenesis, cerebral organoid/GSC models for neuronal/glial–glioma interactions, and GEMMs engineered to express specific tumor antigens for immunotherapy studies. These models would ultimately be agreed upon by the academic and pharmaceutical communities, forming a “credentialed toolbox” used as the baseline for preclinical evidence. After passing this baseline, any biological concept or therapeutic agent would need validation in more sophisticated, patient-relevant models that replicate the complexity of human GBM as discussed in this article. Establishing such standardized preclinical criteria would streamline critical evaluations of manuscripts, funding proposals, and drug development efforts, ensuring a more robust pathway from preclinical research to clinical trials.

Finally, as one envisions the future of tumor and GBM modeling, we must acknowledge that as our understanding of the disease’s complexity rapidly grows, the need for increasingly sophisticated models will only intensify. For example, spatial transcriptomic profiling of GBM samples has recently revealed a five-layered hierarchical arrangement of cellular states that play key roles in organizing tissue architecture (Greenwald et al., 2024; Fazzari et al., 2024, *Preprint*; Meyer et al., 2024; Mathur et al., 2024; Ravi et al., 2022). Given this type of complexity, combined with the heterogeneity of both gliomas and the patients who suffer from them, it seems unlikely that any purely biological model will soon be capable of fully replicating the disease in a truly patient-specific manner. Instead, we speculate that the future of clinically relevant, patient-specific glioma models will likely be found in silico. Although in silico models may have seemed like science fiction just a few years ago, rapid advancements in -omic technologies, personalized medicine, bioengineering, and artificial intelligence (AI) make their development both feasible and imminent (Bhinder et al., 2021; Ritzau-Reid et al., 2023).

Thus, we propose that the next generation of GBM models will involve a complex synthesis of hybrid systems that will integrate AI with preclinical models and clinical trial data to create patient-specific systems. As preclinical models advance, incorporating patient-derived cells, genomics/epigenomics, organoids, and advanced tissue/bioengineering systems that more accurately replicate the tumor microenvironment, they will generate vast amounts of biologically interrelated data. These data will be incorporated into AI-driven platforms, enabling the construction of predictive models that continuously refine their accuracy by integrating multi-omics clinical trial endpoint data (e.g., drug response, toxicity), and clinical observational and populational science data, ultimately permitting algorithms capable of predicting patient-specific responses, providing unprecedented precision in treatment selection. Over time, through iterative processing of such extensive and nonlinear datasets, these AI systems will predict therapeutic outcomes with greater accuracy and generate new biological insights, fundamentally transforming personalized GBM treatment.

**Figure 3.**
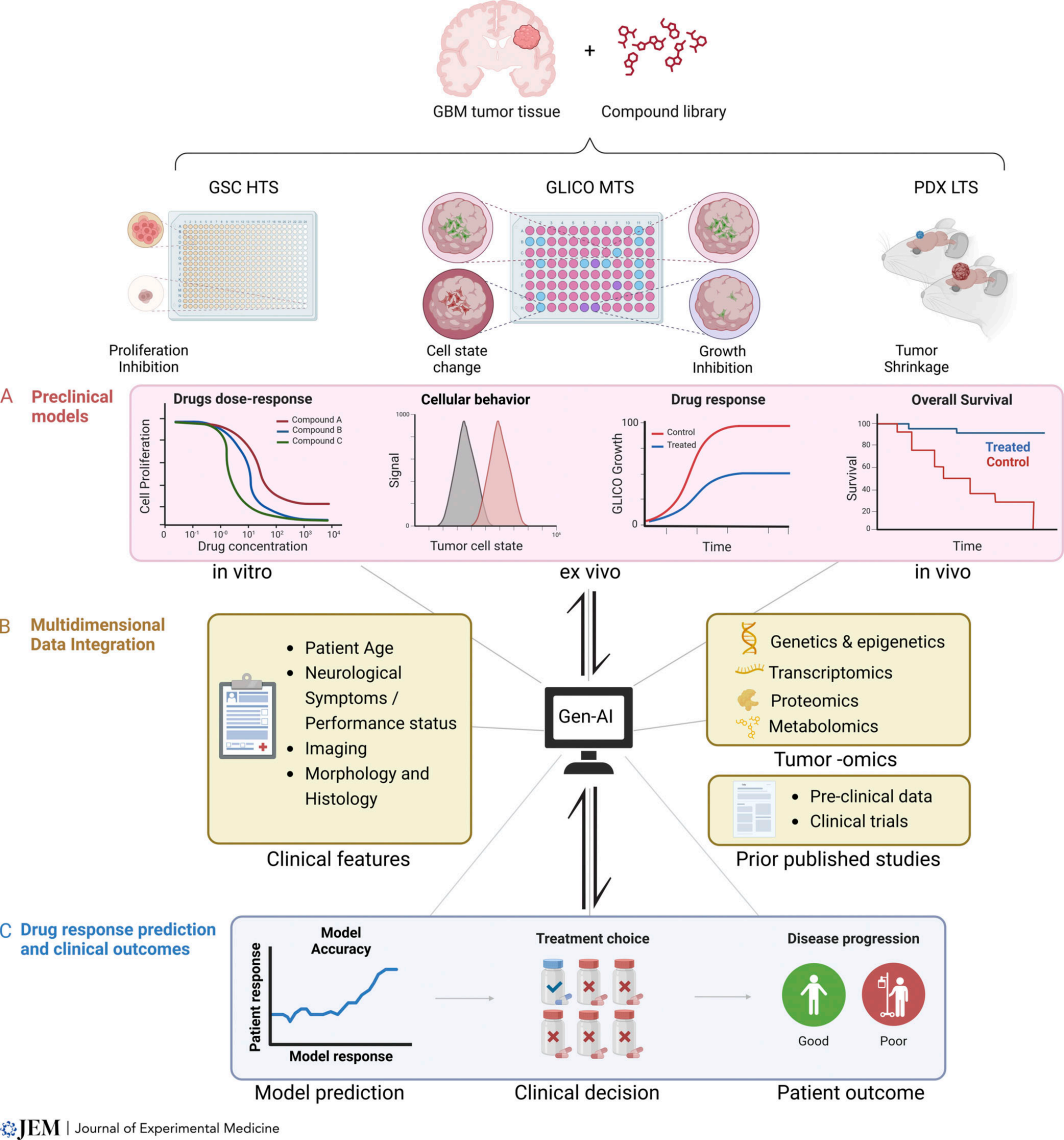
**Generative AI (Gen-AI) in GBM models. (A)** Preclinical data input: Readouts from preclinical models—including cell proliferation, cellular phenotypes, drug response, and survival metrics—are entered into the Gen-AI. **(B)** Multidimensional data integration: AI processes and integrates clinical and molecular data, including patient-specific clinical features (e.g., age, neurological symptoms, and imaging) and tumor-omics (genetics, epigenetics, proteomics, and metabolomics), along with insights from prior published studies and clinical trials. This integration allows the AI to categorize and decode complex model profiles. **(C)** Personalized treatment prediction and model optimization: Based on this comprehensive analysis, AI generates improved, personalized treatment options, linking preclinical model’s predictive drug response to real patient outcomes. The model’s accuracy is continually assessed, allowing for an iterative approach where outputs inform further model refinement, new screening strategies, and enhancements to therapeutic development. HTS, high-througput sequencing; MTS, medium-throughput sequencing; LTS, low-throughput sequencing. Created in BioRender.

Fig. 3 outlines a schematic example of an initial framework for training AI-driven computational models. While historical data archives from legacy models will provide a valuable initial learning dataset, the machine learning platforms will be initially limited by the constraints of the original models. Only new data from the advanced preclinical models discussed earlier will enable AI systems to accurately identify and validate critical vulnerabilities within GBM (Darmanis et al., 2017; Yuan et al., 2018; Neftel et al., 2019; Sankowski et al., 2019; Wang et al., 2019, 2020; Bhaduri et al., 2020; Couturier et al., 2020; Goswami et al., 2020; Wu et al., 2020; Yu et al., 2020; Johnson et al., 2021; Mathewson et al., 2021; Pombo Antunes et al., 2021; Richards et al., 2021; Zhao et al., 2021).

As a side note, it is important to address a potential paradox that will emerge with early efforts to validate the positive predictive value of any preclinical model for identifying clinically active drugs in GBMs. To date, despite over 2,000 clinical trials for malignant gliomas over the past two decades, only one drug (temozolomide) has demonstrated improved survival in GBM. Therefore, in the absence of clinically active drugs, models can currently be validated only for negative predictive value based on previous negative clinical trials. If the lack of predictive models is a reason for the absence of new therapies as we have suggested, then we face a challenge in validating future models. Therefore, any drug showing clinical promise in the future must be retroactively rigorously reassessed through new hybrid models to validate predictive accuracy. Over time, as more drugs demonstrate clinical activity, an iterative process of drug screening and clinical data analysis will refine AI-driven models and enhance their predictive power.

The success of this approach will require the collective will of the research community to work together to meticulously identify, annotate, and publicly communicate the strengths and weaknesses of the chosen biological model systems incorporated into this hybrid system. Consistent annotation, data standardization, publicly accessible computational and clinical outcome datasets, and novel, preferably open-source, AI platforms are essential to enable all interested and expert investigators and computer/software engineers to generate such models. Thus, to ultimately be successful, this iterative system will require a high degree of data and model sharing, communication, and collaboration between teams of basic, translational, computational, and clinical investigators.

## Summary

Preclinical cancer models have been essential for advancing our understanding of cancer biology; however, their limitations in accurately reflecting the complexity of human tumors, particularly gliomas and the patients affected by them, underscore the need for more sophisticated and contextually relevant models. Future glioma models must capture this complexity by incorporating the genomic, epigenomic, and transcriptomic heterogeneity of tumors, along with the unique characteristics of the human brain microenvironment, while still being suitable for high-throughput assays.

The integration of advanced bioengineering, -omics technologies, and AI-driven computational and in silico models offers significant promise for developing clinically relevant, patient-specific models in the near future. These innovations will be crucial for optimizing therapeutic screening and, ultimately, improving outcomes for patients suffering and dying from therapeutically resistant tumors like malignant GBMs.
